# Epigenetic signatures of attachment insecurity and childhood adversity provide evidence for role transition in the pathogenesis of perinatal depression

**DOI:** 10.1038/s41398-020-0703-3

**Published:** 2020-02-03

**Authors:** Thalia K. Robakis, Siming Zhang, Natalie L. Rasgon, Tongbin Li, Tao Wang, Marissa C. Roth, Kathryn L. Humphreys, Ian H. Gotlib, Marcus Ho, Arineh Khechaduri, Katherine Watson, Siena Roat-Shumway, Vena V. Budhan, Kasey N. Davis, Susan D. Crowe, Katherine Ellie Williams, Alexander E. Urban

**Affiliations:** 1 Stanford University Department of Psychiatry and Behavioral Sciences, Stanford, CA USA; 2 Stanford University Department of Genetics, Stanford, CA USA; 3 AccuraScience, LLC, Johnston, IN USA; 4 Vanderbilt University Department of Psychology, Nashville, TN USA; 5 Stanford University Department of Psychology, Stanford, CA USA; 6 Fred Hutchinson Cancer Research Center, Seattle, WA USA; 7 Palo Alto University Graduate School of Psychology, Palo Alto, CA USA; 8 Stanford University Department of Obstetrics & Gynecology, Stanford, CA USA

**Keywords:** Depression, Clinical genetics, Predictive markers

## Abstract

Early life adversity and insecure attachment style are known risk factors for perinatal depression. The biological pathways linking these experiences, however, have not yet been elucidated. We hypothesized that overlap in patterns of DNA methylation in association with each of these phenomena could identify genes and pathways of importance. Specifically, we wished to distinguish between allostatic-load and role-transition hypotheses of perinatal depression. We conducted a large-scale analysis of methylation patterns across 5 × 10^6^ individual CG dinucleotides in 54 women participating in a longitudinal prospective study of perinatal depression, using clustering-based criteria for significance to control for multiple comparisons. We identified 1580 regions in which methylation density was associated with childhood adversity, 3 in which methylation density was associated with insecure attachment style, and 6 in which methylation density was associated with perinatal depression. Shorter telomeres were observed in association with childhood trauma but not with perinatal depression or attachment insecurity. A detailed analysis of methylation density in the oxytocin receptor gene revealed similar patterns of DNA methylation in association with perinatal depression and with insecure attachment style, while childhood trauma was associated with a distinct methylation pattern in this gene. Clinically, attachment style was strongly associated with depression only in pregnancy and the early postpartum, whereas the association of childhood adversity with depression was time-invariant. We concluded that the broad DNA methylation signature and reduced telomere length associated with childhood adversity could indicate increased allostatic load across multiple body systems, whereas perinatal depression and attachment insecurity may be narrower phenotypes with more limited DNA methylation signatures outside the CNS, and no apparent association with telomere length or, by extension, allostatic load. In contrast, the finding of matching DNA methylation patterns within the oxytocin receptor gene for perinatal depression and attachment insecurity is consistent with the theory that the perinatal period is a time of activation of existing attachment schemas for the purpose of structuring the mother–child relationship, and that such activation may occur in part through specific patterns of methylation of the oxytocin receptor gene.

## Introduction

Early life adversity is a risk factor for psychiatric illness in adulthood^1^. In particular, the perinatal period is a time of high risk for episodes of depression^2^, and history of childhood adversity is a major risk factor for perinatal depressive episodes^3–5^.

Early life adversity has pleiotropic effects, behavioral and physiological, on multiple body systems^6^, including those important for both psychiatric and reproductive function. History of abuse in childhood is known to alter hypothalamic-pituitary-adrenal axis responses^7^ and to increase the amplitude of stress responses precipitated by gonadal steroid fluctuation^8^. Childhood sexual abuse is associated with earlier menarche, and childhood physical abuse with later menarche^9^. Exposure to childhood maltreatment, particularly emotional abuse, has also been associated with reduced concentrations of oxytocin, a hormone that plays prominent roles in parturition and mother-infant bonding, in cerebrospinal fluid^10^.

### Allostatic load versus role transition in risk for perinatal depression

The mechanisms by which early life experiences contribute to risk for adult psychopathology are complex and not yet fully understood. Chronic stress imposed by early life adversity has been posited to result in increased total allostatic load^11^, a body-wide burden imposed by the chronic expression of physiological adaptations to stress, which increases risk for mood disorders across the lifespan. It is possible that the association of perinatal depression with childhood adversity is the result of increased allostatic load in individuals with histories of trauma.

Alternatively, depression in the perinatal period may be related to specific psychological responses to the role transition to parenthood. This role transition requires the new parent to draw on a behavioral and emotional blueprint for caregiving that relies heavily on the individual’s past life experiences^12^. Failure of the new parent to identify an adequate internal blueprint for caregiving, such as might occur in individuals who did not receive appropriate caregiving in their own childhoods, could be associated with role confusion and risk for depression^13^. The findings that adult attachment style^14–16^ and parenting style experienced in childhood^17^ strongly predict perinatal depression are consistent with this second potential causal pathway.

We postulated that the molecular pathways associated with each of these explanations would differ.

### Molecular pathways from early life adversity to adult perinatal depression

The molecular pathways by which early life experiences are transduced into lasting physiological and psychiatric outcomes are a subject of ongoing investigation. A foundational discovery in this area was the demonstration that early life experiences could be inscribed on the genome via methylation of CpG dinucleotides, and thereby affect personality and behavior in adulthood^18,19^. Since that demonstration, human and animal evidence has accumulated supporting alterations in patterns of DNA methylation as a link between early life adversity and adult psychopathology^20–24^.

With respect to perinatal depression, two regions of alternative DNA methylation in association with postpartum depression have been previously identified in leukocytes, resulting from a screen that was designed to identify DNA methylation effects of estrogen activity^25,26^. Later work from this dataset using a candidate-gene approach also identified alterations in methylation density within the oxytocin receptor gene in association with postpartum depression^27^. Other groups have also reported associations of oxytocin receptor DNA methylation with perinatal depression, in tissues such as saliva^28^, whole blood^29^, and placenta^30^.

While initial work on DNA methylation focused on selected candidate genes, behavioral-level effects are likely to be produced by coordinated changes in expression and function across large suites of RNA and protein products. The advent of next-generation sequencing and advanced data analysis techniques has made it possible to explore large regions of the genome with unbiased searches. While several investigators have reported the results of unbiased searches for alterations in DNA methylation in association with childhood trauma^20–23^, only one study has done so thus far for postpartum depression, examining DNA from maternal T lymphocytes, and reporting no significant results^31^, possibly due to a smaller N of 38.

### Approach

We postulated that, consistent with allostatic load theory, early life adversity would be associated with epigenetic changes to a broad array of genes, some of which might also have relevance to perinatal depression. Overlap in the genomic regions that are epigenetically sensitive to early life adversity with those regions in which epigenetic alteration is associated with perinatal depression could indicate biological pathways that may be of particular importance in the determination of vulnerability to perinatal depression conferred by early life adversity.

Therefore, we conducted unbiased explorations of DNA methylation density across a large selection of regions that represent the most epigenetically active portion of the genome^32^. We conducted separate explorations to examine associations of DNA methylation density with childhood adversity and with antenatal and postpartum depression. We examined buccal epithelial cells, an accessible peripheral tissue of high cell type homogeneity^33^, shared embryonal origin with neural cells^34^, and established utility for DNA methylation studies^33^. As an additional molecular proxy for allostatic load, we measured telomere length^35^.

As we were interested in identifying mediating pathways between early life adversity and perinatal depression, we also explored DNA methylation patterns in association with insecure attachment style, a powerful risk factor for postpartum depression^14,15^ that has been hypothesized to mediate the effects of childhood adversity on risk for depression in adulthood^36^ and to be critical for shaping maternal behavior^12^. We reasoned that attachment insecurity could be an important precedent of perinatal depression in the role transition theory discussed above.

Given extensive previous work on and interest in the oxytocin receptor and its clear relevance to both attachment security^37,38^ and psychiatric morbidity in the perinatal period^27–30^, we also examined patterns of methylation density in association with perinatal depression, childhood trauma, and attachment insecurity within this candidate gene.

We recognize that our antecedent variables of interest, namely early life adversity and attachment style, as well as their putative molecular proxies, could have complex relations to each other. Insecure attachment might be a mediator of the association between early life adversity and perinatal depression, as previously hypothesized^36^; alternatively it could represent a distinct pathway to perinatal depression. These explanations are not necessarily mutually exclusive, with partial mediation also being a possibility.

If attachment insecurity were to mediate the effect of childhood trauma on perinatal depression, we could expect to find similar or highly overlapping DNA methylation signatures for childhood trauma, insecure attachment style, and perinatal depression. If attachment insecurity and childhood trauma were distinct antecedents of perinatal depression, we could expect distinct DNA methylation signatures for each of them. In the case of partial mediation, we could expect to detect partial overlap in methylation patterns in a limited subset of genes or regions.

## Materials and methods

### Technical advances

In this work we have been able to implement several technical advances in comparison to previous work in the field. While published studies on this topic to date^25,31^ have used Illumina Infinium BeadChip arrays, we used a next-generation sequencing approach, which is less susceptible to stochastic variation than the fluorescence intensities used in the array, and therefore yields a more reliable estimate of small differences in methylation density. In addition, the sequencing based assay allows readout of many more individual CpGs than the arrays (i.e., several million CpGs per proband when using sequencing compared to several hundred thousand when using arrays).

We have also developed a tailored approach to managing the problem of multiple hypotheses in the context of epigenomic research. A detailed, locus-level exploration of candidate genes from our dataset revealed a tendency for loci in which methylation density is associated with attachment insecurity to form spatial clusters along the DNA. This is consistent with a large body of research that documents spatial clustering among functionally associated CpGs^39^, and with the generally accepted acknowledgement that some of the primary functions of DNA methylation, such as blocking access to the transcription machinery, depend on the accumulation of multiple methyl groups over a stretch of DNA^40,41^, thus contributing to the clustered patterns of methylation that are observed over the genome. As others have proposed^42^, we suggest that this spatial clustering can be used as an additional indicator of biological significance beyond the traditional statistical reliance on the observed strength of the association.

Finally, we reasoned that the variables of interest were generally continuous rather than discrete. Therefore, rather than imposing artificial case-control distinctions, we computed correlation coefficients between methylation density and the clinical variables of interest (childhood trauma, depressive symptoms, and attachment insecurity). These procedures increased our power to detect significant effects within a moderate sample size. See sections below on ‘DNA Methylation Analysis’ and ‘Statistical Analysis’ for procedural details.

### Participants

This study was approved by the Stanford University Institutional Review Board for Human Subjects Research, and all study participants completed informed consent prior to study participation. Power analysis for the effect of attachment insecurity on postpartum depression, based on our previously published pilot dataset^15^, indicated a large effect size (Cohen’s *d* = 0.94), which suggested a minimum total sample size of 38 to detect the effect with *p* = 0.05. The clinical sample was a cohort of 124 healthy pregnant women recruited from local obstetric clinics, community postings, and the Stanford University reproductive psychiatry clinic, between August 2013 and September 2016. Recruitment through the psychiatric clinic allowed us to oversample for women with histories of mood disorder. Inclusion criteria were age at least 18, uncomplicated singleton pregnancy, and the ability to complete the interview and self-report measures in English. Exclusion criteria were multiple or highly medically complicated pregnancy, or insufficient English to participate. The epigenetic sample was recruited in part from the clinical sample population (44 participants) and in part from a second study with equivalent recruitment criteria and follow-up procedures (16 participants). Intake interviews occurred between 27 and 40 weeks gestation (mean 33.3 weeks, SD 4.1 weeks). Six samples were discarded for poor sequencing quality, resulting in a total sample size of 54 women for the DNA methylation study. See Supplementary Fig. 1 for study flowchart.

### Measures

Each participant completed a full psychiatric interview (SCID^43^) in the third trimester of pregnancy, at which time the Attachment Style Questionnaire (ASQ^44^) and Edinburgh Postnatal Depression Scale (EPDS^45^) were administered, and a buccal swab (Mawi DNA Technologies) was provided for DNA analysis. The Childhood Trauma Questionnaire (CTQ^46^) was administered at intake for 16 women, and was completed at a later time for the remaining women who did not receive it at intake. Participants were asked to complete the EPDS on a monthly basis for six months postpartum. Overall there was a 77.8% rate of return for all follow-up surveys (578 surveys completed out of 744 surveys sent). All women did provide at least one follow-up survey response.

The EPDS is a 10-item self-report measure of depressive symptoms that was designed for use with perinatal women. It is used for both screening and severity assessment and has been validated cross-culturally^47^. When defining postpartum-depressed versus postpartum-nondepressed, we chose a stringent cutoff of 12 rather than the more liberal cutoff of 9 that is often used in clinical screening, because specificity was more important than sensitivity for the purpose of defining study groups^48^.

The ASQ is a 40-item self-report measure of attachment insecurity that assesses the individual’s attitudes towards relationships with others. The ASQ has been validated by demonstrating patterns of association with other measures of adult attachment^49^. It has been used previously in pregnant women^50,51^ and in the study of postpartum depression^52^. The ASQ has five subscales: Confidence, Need for Approval, Relationships as Secondary, Discomfort with Closeness, and Preoccupation with Relationships. These subscales have been mapped onto the subcategories of anxious and avoidant attachment used by other measures^49^. For this paper we computed an adjusted total attachment insecurity score in which the Confidence score was subtracted from the sum of the scores of the four other subscales. We did this based on our previous work showing that both anxious and avoidant attachment subtypes confer similar levels of risk for postpartum depression^15^.

The CTQ is a 25-item self-report measure that yields subscales of five categories of childhood trauma: physical abuse, emotional abuse, sexual abuse, physical neglect, and emotional neglect. We used a combined total of all subscales for the epigenetic and telomere analysis initially, and also conducted subsequent analyses of each of the five subscales with respect to their relation to telomere length.

### DNA methylation analysis

Genomic DNA was extracted from buccal swabs using Blood & Tissue Culture DNA mini kit (Qiagen). Library construction was performed using SeqCap Epi Enrichment System (Roche). In brief, 1 μg of genomic DNA was sonicated (Covaris) to generate fragments 180–220 bp in size. Following fragmentation, DNA was used to construct library as described in the KAPA library Preparation Kit Illumina platforms (KAPA biosystems). After library construction, DNA libraries were bisulfite converted and purified using the EZ DNA Methylation Lightning Kit (Zymo Research). Bisulfite converted samples libraries were amplified using LM-PCR. Amplified bisulfite converted samples and SeqCap Epi probes were hybridized for 64–72 h at 47 °C. Captured DNA samples were washed and recovered, followed by amplification of the capture DNA samples using LM-PCR. Amplified samples were purified using Agencourt AMPure XP Beads (Beckman Coulter). Final libraries were checked for quality on a Bioanalyzer DNA 1000 chip (Agilent).

Bisulfite converted libraries captured by SeqCap Epi CpGiant Probes kit (the size of target regions is 80.5 Mb with > 5.5 million CpGs) of all the samples were sequenced at the Functional Genomics Facility (Stanford University) on Illumina HiSeq 4000 platform by 2 × 150 paired-end sequencing with an average of 70 million reads generated for each sample. After trimming the adapters and low-quality ends by Cutadapt^53^, the reads were mapped to human RefSeq genome hg19 using Bismark (Version 0.16.3) (95) with an average unique mapping rate of 65.0% of all the samples. Duplicates were removed by the deduplicate_bismark script in Bismark. Only one copy of the overlapping parts in the middle of paired-end reads was retained after clipping the read with the lower average quality in the overlap region by the “clipOverlap” tool in bamUtil (Version 1.0.14). On-target read rate and coverage were calculated by Qualimap (Version 2.1)^54^. Six samples with <3x average on-target coverage were discarded. The average on-target read rate of all retained samples was 87.49% and average on-target coverage was 27.6×.

The methylation ratio for each CpG was extracted by the bismark_methylation_extractor script in Bismark. For each sample, only CpGs with at least ten reads covering them were included in the downstream analysis.

To determine bisulfite conversion efficiency of each captured library by SeqCap Epi CpGiant Probes kit, unmethylated genomic DNA of Enterobacteria phage lambda was added and processed together with the sample DNA in the same tube throughout the library preparation and sequencing. Each kit contains probes to capture the lambda genomic region from base 4500 to 6500. The sequencing reads were aligned to the phage lambda genome (GenBank Accession NC_001416) by Bismark and the bismark_methylation_extractor script was used to extract the methylation ratio for CpGs of the lambda genome. The reads aligned to the captured region of the lambda genome can be used to calculate the overall conversion efficiency as follows: conversion rate = 1−(sum(C_count)/sum(CT_count)). The average of bisulfite conversion rate of all the samples was 99.3%.

### Statistical analysis

We developed a sliding-window based statistical analysis approach similar to the swDMR method previously described^42^, with the key difference that instead of identifying differentially methylated regions (DMRs) that differ significantly between two groups of subjects as swDMR1 does, we identified methylation regions that are significantly associated with a continuous or quantitative clinical variable, for the analysis of depressive symptoms, attachment style, and history of trauma. The method we developed is as follows: starting from every single CpG locus, a window is defined if more than 5 CpGs are identified within a 1000 bp span. For each window identified, mean methylation density is calculated for each patient. If methylation density data of CpGs in a window are all missing (NA) for a patient, the mean methylation density for this patient is imputed as 0. For the clinical variables, each missing value (NA) is imputed using the mean value for the variable from all other patients. Association analysis is conducted by computing the Pearson’s correlation coefficient (PCC) between the values of the continuous clinical variable and the mean methylation densities. The *P* value is derived from the PCC using Fisher’s Z transformation test. Benjamini-Hochberg^55^ method is then applied to calculate false discovery rates (FDRs) based on the *P* values. Significant regions are extended by merging overlapping CpGs among nearby windows. The *P* values and FDRs are then re-calculated to select new windows. This process is repeated iteratively until no neighboring significant regions within a 100 bp distance can be merged. The method described above is implemented using locally developed Python code. Selected cutoffs for positive results were *P* < 0.05 and FDR > 0.2. Requests for access to source code should be directed to Accura Science, LLC (info AT gdb.accurascience.com).

For the identification of differentially methylated regions related to history of depression, a binary variable, a standard DMR analysis was used. Methylation ratios for CpGs, defined as methylated reads divided by total reads, were called by Bismark. Differentially methylated regions were identified using Metilene using a minimum cutoff of 0.1 mean methylation difference between groups. *P*-value and FDR cutoffs for screening were as above, although in practice, all regions that simultaneously fulfilled the p, FDR, and mean methylation difference criteria had *P* < 10^−7^ and FDR < 0.05 (Table 4).

### Genomic annotation for significant methylation regions

The script annotatePeaks.pl included in HOMER3 was invoked to annotate significant methylation regions into one of the following categories: exon, intron, promoter, TTS, 5′ UTR, 3′ UTR, intergenic and non-coding. In this analysis, a promoter region was defined to be the region between −1 kb and +100 bp of a known gene’s TSS. Characterization of overlaps across significant methylation regions for different clinical variables: BEDTools4 was used to search overlapped regions among significant methylation regions identified across clinical variables. We call two significant methylation regions overlapping if they are overlapped by more than a certain percentage overlap cut-off (POC) value for each of the two regions.

### Molecular function classification

The online molecular function classification tool Panther^56^ was used to explore the molecular function classifications for the results obtained.

### Oxytocin receptor gene methylation analysis

The capture region included a region of 5016 bp of the oxytocin receptor gene (*OXTR*), which included 151 distinct CpG sites. This region covered most of the gene sequence, including all four exons and excluding only a portion of the last large intron. Using SPSS version 25, individual Pearson correlations were run between methylation density at each of the 151 CpG sites and scores on the clinical variables of interest. For the analysis of mediation by *OXTR* methylation of the association between attachment style and depression, a principal components analysis using SAS was done to reduce the ten loci that comprise the oxytocin receptor methylation pattern based on attachment style into four principal components. Each principal component was then evaluated as a mediator for the relationship between total ASQ and antenatal EPDS and the relationship between total ASQ and postnatal EPDS using the MacArthur approach. Additionally, each locus was evaluated as an individual mediator for these two relationships using the MacArthur^57^ approach.

### Telomere analysis

Average relative telomere length was measured by quantitative PCR using a method adapted from the original published method by Cawthon^58,59^ and expressed as the ratio of telomere abundance vs. a single copy gene (human b-globin) abundance (T/S ratios). The T/S ratio for each sample was measured twice. When the duplicate T/S value and the initial value varied by more than 7%, the sample was run a third time and the two closest values were reported. The average coefficient of variation (CV) of this study was 3.0%. Lab personnel who performed the telomere length measurement were blind to all participant characteristics. One outlier with inexplicably short telomere length result of 0.39 was removed from the final analysis.

## Results

### Demographics

Nearly all participants were married or partnered and 60% were primiparous. Participants, on average, had high levels of educational attainment, and primarily endorsed Caucasian, East Asian, and South Asian ethnic identities, consistent with the population characteristics of the San Francisco Bay Area’s Peninsula region. Over one-third (36%) had past history of mood disorder, consistent with the strategy of oversampling for psychiatric history via recruitment through the reproductive psychiatry clinic. 23.6% were positive for depression in the postpartum period (EPDS > 12 at either month 1 or month 2), with rates of postpartum depression being 16.6% in those with no psychiatric history and 36.6% in those with a history of mood disorder as determined by SCID at interview. See Table 1 for full demographics.

**Table 1:** Sample demographics.

	Epigenetic sample (*N* = 54)					Clinical sample (*N* = 124)				
	All	No mood disorder (28)		Mood disorder (26)		All	No mood disorder (79)		Mood disorder (45)	
Age at intake (mean, SD)	32.33 (4.40)	32.39 (4.18)		32.27 (4.72)		32.31 (4.79)	31.73 (4.51)		33.31 (5.16)	
		Number	%	Number	%		Number	%	Number	%
Marital status										
Single	1	0	0.0	1	3.8	3	2	2.5	1	2.2
Married	27	25	89.3	2	7.7	115	74	93.7	41	91.1
Partnered	26	3	10.7	23	88.5	6	3	3.8	3	6.7
Parity										
0	37	21	75.0	16	61.5	75	48	60.8	27	60.0
1	15	5	17.9	10	38.5	36	21	26.6	15	33.3
2 or more	1	1	3.6	0	0.0	13	10	12.7	3	6.7
Education										
No HS diploma	0	0	0.0	0	0.0	2	1	1.27	1	2.22
HS or GED	3	1	3.6	2	7.7	2	1	1.27	1	2.22
Some college	4	2	7.1	2	7.7	18	13	16.46	5	11.11
Bachelor’s	24	12	42.9	12	46.2	41	23	29.11	18	40.00
Graduate schooling	23	13	46.4	10	38.5	61	41	51.90	20	44.44
Employed at intake										
Yes	42	21	75.0	21	80.8	42	50	63.29	32	71.11
No	12	7	25.0	5	19.2	82	29	36.71	13	28.89
Ethnicity										
Caucasian	35	17	60.7	18	69.2	71	42	53.16	29	64.44
East Asian	6	3	10.7	3	11.5	18	13	16.46	5	11.11
South Asian	2	2	7.1	0	0.0	10	9	11.39	1	2.22
Hispanic	3	2	7.1	1	3.8	13	8	10.13	5	11.11
African-American	1	0	0.0	1	3.8	1	0	0.00	1	2.22
Multiracial	6	4	14.3	2	7.7	11	7	8.86	4	8.89
Psychiatric diagnosis										
Unipolar depression				25	96.2				37	82.22
Bipolar disorder^a^				1	3.8				8	17.78
Depression, EPDS ≥ 12 Antenatal										
Yes	6	2	7.4	4	14.8	20	9	11.4	11	24.4
No	48	25	92.6	23	85.2	104	70	88.6	34	75.6
Postnatal										
Yes	9	2	8.0	7	28.0	29	13	16.6	16	35.6
No	41	23	92.0	18	72.0	94	65	83.3	29	82.9
CTQ score (mean, SD)	35.71 (14.7)	32.14 (8.8)		39.12 (18.3)		37.78 (12.8)	37.04 (12.8)		39.09 (12.7)	
ASQ score (mean, SD)	60.81 (32.8)	50.11 (26.7)		71.52 (35.2)		62.91 (29.2)	56.13 (24.7)		74.82 (32.9)	

^a^Sensitivity analysis revealed no effect of mood disorder history on the association between ASQ and postnatal EPDS, therefore we elected to keep participants with bipolar disorder in the dataset. In all analyses involving mood disorder history, bipolar and unipolar disorders are coded separately.

### Associations among attachment insecurity, childhood trauma, and perinatal depression

**Fig. 1:**
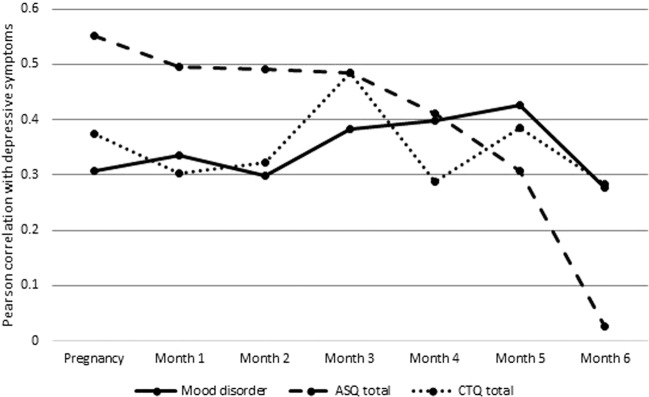
Correlations between baseline characteristics and depressive symptoms at each time point for the clinical sample (*N* = 124). Pearson correlations between Edinburgh postpartum depression scale score at and each of mood disorder history, total childhood trauma score, and total attachment insecurity score are plotted at each of seven time points, beginning in late pregnancy and ending at 6 months postpartum. Insecure adult attachment style is of particular importance to mood during pregnancy and the early postnatal period compared to 4 months or more postpartum. Supplementary Table 1. Multiple regression showing attachment insecurity and childhood trauma as predictors of depressive symptom severity.

Participants in the clinical sample provided monthly EPDS scores for 6 months postpartum. Correlations between baseline measures of attachment insecurity, childhood trauma, and mood disorder and depressive symptom severity were plotted at each of the monthly assessments. We found that attachment insecurity displayed an unusually strong correlation with depression score in pregnancy and in the early postpartum period, but that after about 4 months postpartum the strength of this association decayed. Anxious and avoidant subtypes of attachment showed similar correlation strengths with depressive symptoms (data not shown). For childhood trauma and history of mood disorder, the association with depressive symptom severity was relatively flat. By 6 months postpartum, childhood trauma, mood disorder history, and attachment insecurity all had similar correlation strengths with depressive symptom severity. (Fig. 1). To further explore the effects of time since delivery, we were able to obtain follow-up data on depressive symptoms at 3– 5 years postpartum from 93 of the women in the clinical sample, using the CESD, a more general measure of depressive symptoms. Over this longer timeframe, baseline ASQ also showed a decay in the strength of correlation with depressive symptoms (Pearson correlation between ASQ at intake and CESD at intake = 0.555, and between ASQ at intake and CESD 3–5 years later = 0.371, *P* < 0.001 for both) whereas mood disorder history and CTQ scores remained similar in the magnitude of their association with CESD, regardless of time point (mood disorder history:intake CESD, 0.258, *P* = 0.013; mood disorder history: 3–5 y CESD, 0.264, *P* = 0.011; intake CTQ:intake CESD = 0.335, *P* = 0.001; intake CTQ:3–5 y CESD = 0.315, *P* = 0.003).

**Fig. 2:**
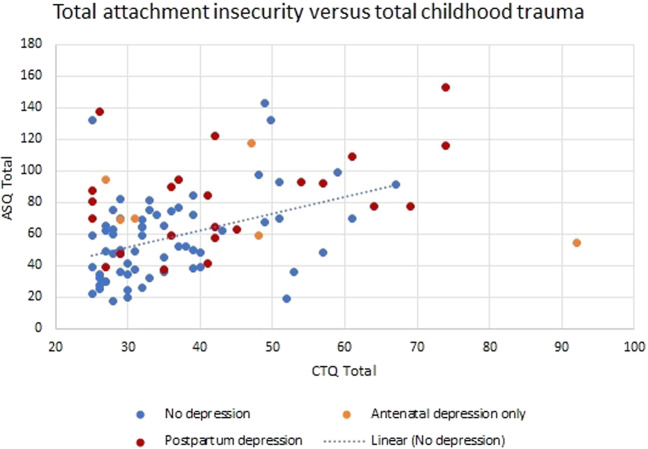
Scatterplot of CTQ and ASQ scores in the clinical sample (*N* = 124). Perinatal depression is defined as EPDS > 12 at any of the pregnancy, Month 1, or Month 2 time points. Pearson correlation for full sample = 0.391, *P* < 0.001.

Because attachment insecurity is known to be more common in individuals with childhood adversity, we also plotted the association between CTQ and ASQ scores in our sample. We found that attachment insecurity was correlated, but by no means synonymous, with childhood trauma (Pearson correlation 0.391, *P* < 0.001; Fig. 2). Multiple regression analysis with depressive symptoms as the dependent variable showed that only ASQ retained significance when CTQ was included in the model, and no interaction effect was found (Supplementary Table 1).

### DNA methylation density screen

We identified 1580 regions of interest in which DNA methylation density was associated with CTQ score; three regions in which DNA methylation density was associated with ASQ score, one region in which DNA methylation density was associated with antenatal EPDS score, and five regions in which methylation density was associated with postnatal EPDS score, using our chosen less stringent cutoff of FDR < 0.2. Using a more stringent cutoff of FDR < 0.05, we found 162 regions associated with CTQ score, one region associated with ASQ score, 0 regions associated with antenatal EPDS, and two regions associated with postnatal EPDS. As our interest was to identify broad categories of gene function and to look for overlap among the identified subsets of genes, additional analyses were conducted on the wider result set.

**Fig. 3:**
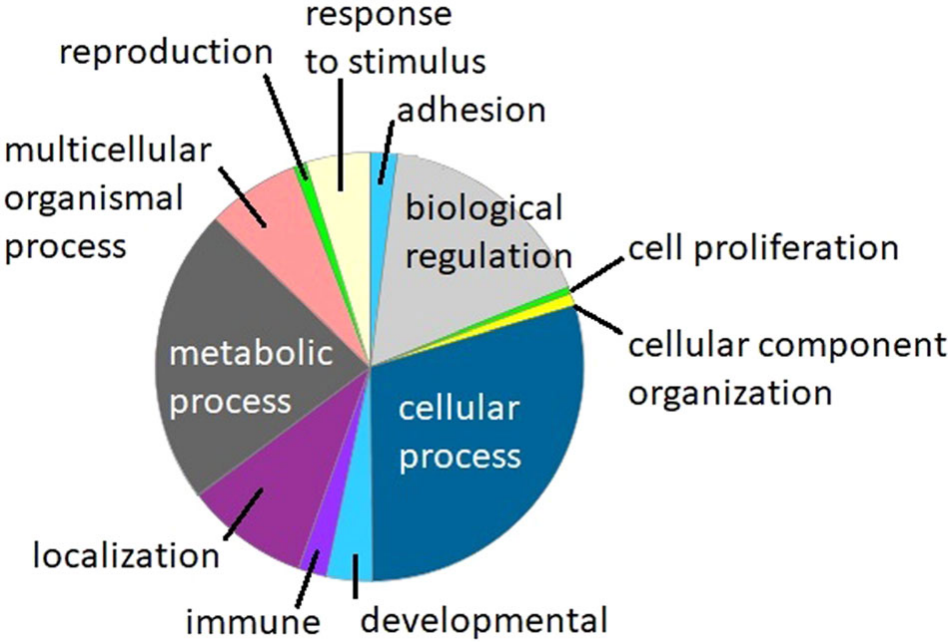
Panther molecular function classification of genes containing regions where methylation density is significantly associated with childhood adversity score. (*N* = 54).

The 1580 regions identified in association with CTQ score were enriched for promoter localization, with 38.9% being localized to promoter regions. Panther molecular function classification analysis^56^ suggested that most of the associated genes coded for protein products with metabolic, cellular process, or regulatory functions (Fig. 3, and see Supplementary Table 2 for full list of results). An enrichment analysis of these results suggested modest enrichment over statistical expectation of genes with protein products involved in cytoskeletal organization, transcription, and metabolic processes (Supplementary Table 3).

The much smaller list of results identified in association with ASQ score and antenatal and postnatal EPDS score respectively were also promoter-localized in four out of nine cases. Of the nine results, two were associated with noncoding regions of DNA. Among the other seven, two were associated with ASQ score and had roles in growth and cell cycle regulation; one was associated with antenatal depression and had a metabolic function; and four were associated with postnatal depression, of which three had binding functions and one a catalytic activity (metalloprotease) function. The full list of these results is presented in Supplementary Table 4.

### Overlap among genomic regions where methylation density is associated with CTQ and postnatal EPDS respectively

Of the five regions where methylation density was associated with postnatal EPDS, one of them, a region of the gene *PLEKHA7*, was also among the 1580 results in the CTQ screen. Association of methylation density in this region with CTQ and postnatal EPDS scores respectively is shown in Supplementary Fig. 2.

### Oxytocin receptor methylation density in association with perinatal depression, attachment style, and trauma history

Within the clinical sample, attachment style was associated with increased risk for perinatal depression in an unusually temporally dependent manner, such that assumed conferred risk was very high during pregnancy and up to 3 months postpartum, and dropped precipitously thereafter. This is in contrast to the findings for childhood trauma and mood disorder history, for which the strength of the association with depressive symptoms remained constant throughout the time period examined (Fig. 1).

**Fig. 4:**
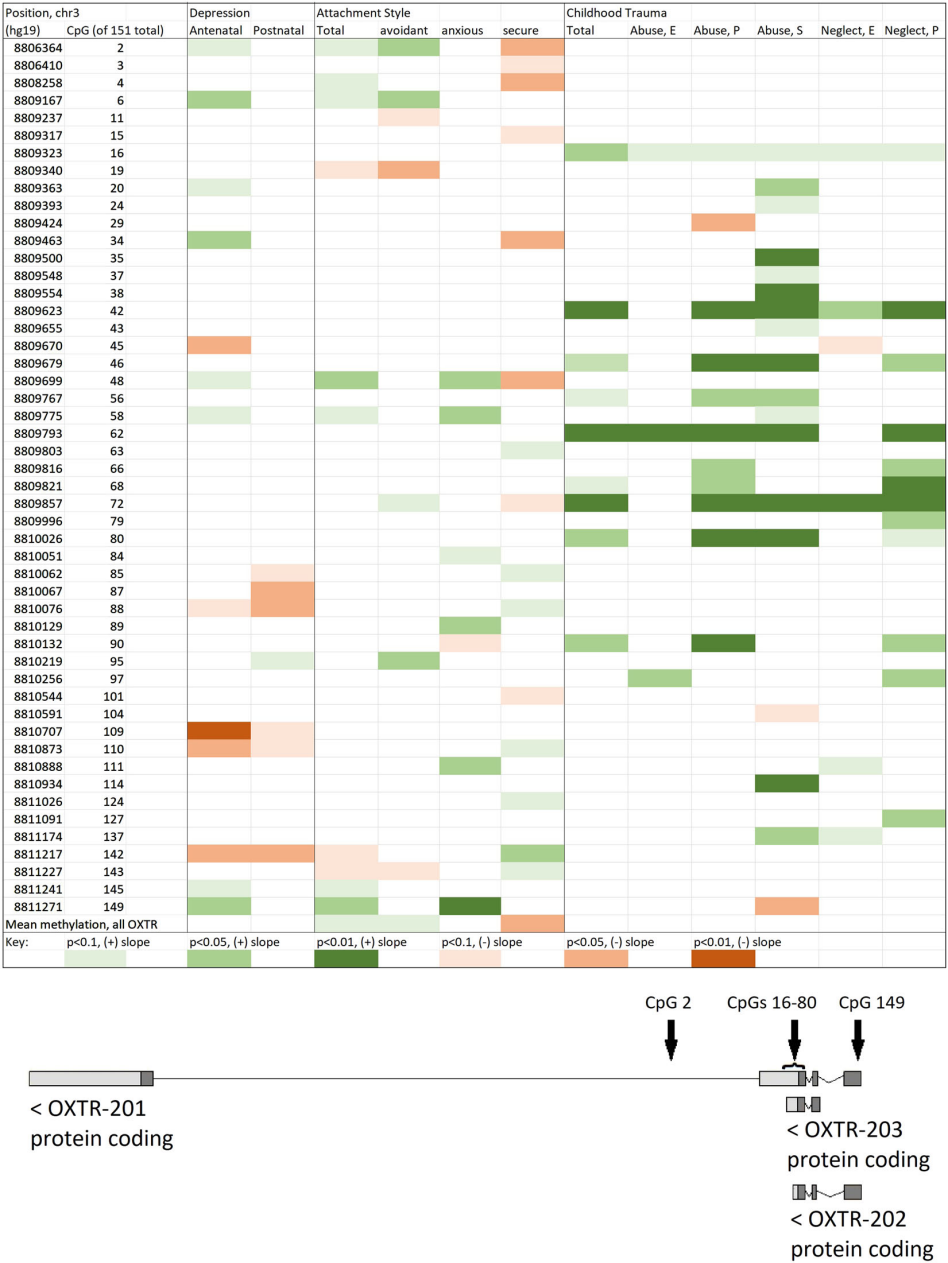
Correlations of DNA methylation density in *OXTR* with perinatal depression, attachment style, and childhood trauma. (*N* = 54) Methylation patterns associated with insecure attachment style (both anxious and avoidant) show similarity to those in antenatal depression, with the inverse pattern observed in association with secure attachment. Childhood trauma shows a distinct pattern of methylation within *OXTR*.

The genomic region captured in our dataset included 151 CpG dinucleotides within *OXTR* (locations shown schematically in Fig. 4). We computed Pearson correlations between methylation density at each of these CpG sites and scores on each of the clinical scales of interest, including all subscales of the ASQ and CTQ. Results of these analyses are presented in Fig. 4. (CpG dinucleotides where no significant association was found with any clinical scale have been omitted from the figure).

Thirteen CpG sites were found in which methylation density was significantly associated with antenatal depression, and four of these plus three alternative sites with postnatal depression. (It is possible that the larger number of results obtained for antenatal depression is related to the fact that the DNA sample was obtained contemporaneously with the depression measure at this time point).

Of these 13 sites, ten also showed associations of the same sign with one of the ASQ subscales showing insecure attachment, or of the opposite sign with the subscale measuring secure attachment. Interestingly, mean methylation density over the entire captured gene region of *OXTR* was positively associated with the insecure subscales of the attachment measure, and negatively associated with the secure subscale. A mediation analysis was run to determine whether methylation density at these ten sites mediated the association between ASQ and depression scores, but mediation was not found.

The CTQ and its subscales showed a distinct pattern of associations with methylation density within *OXTR*, one that was largely nonoverlapping with the pattern observed in association with perinatal depression. Mean methylation density over the entire captured region of *OXTR* was not significantly associated with either total CTQ or with any of the CTQ subscales.

### Telomere analysis

Telomere length was significantly negatively associated with history of emotional abuse (rho = −0.296, *P* = 0.048) and physical abuse (rho = −0.297, *P* = 0.048) in childhood (Supplementary Fig. 3). Trend-level associations were found for the other CTQ subscales and for total CTQ score, but these did not reach statistical significance. Telomere length was also positively associated with educational attainment (rho = 0.382, *P* = 0.004). No association of telomere length with attachment insecurity, depressive symptoms, age, or history of mood disorder was observed.

## Discussion

The purpose of this study was to identify molecular processes underlying the connections between early life adversity, attachment insecurity, and perinatal depression.

Depression in the perinatal period occurs in a context that is distinct from that of depression at other times in life. The transition to parenthood and the taking on of caregiver responsibilities represent a challenge unique to this period. This ‘role transition’ hypothesis is a critical construct in interpersonal psychotherapy for perinatal depression^13^. Women with insecure attachment styles may face particular difficulty with this transition. Our finding that attachment insecurity is most strongly related to depression in the early perinatal period, from late pregnancy through the third month postpartum, supports this hypothesis. The accumulation of allostatic load^11^ is a formative hypothesis for the explanation of the association between childhood adversity and depression in adulthood, but would not be expected to be specific to the perinatal period.

The nature of the association between childhood adversity and adult attachment style is not fully understood. Childhood adversity can be related to attachment insecurity, but is not identical to it. Some individuals who experience childhood adversity prove resilient and go on to develop fulfilling and harmonious interpersonal relationships as adults, whereas others are vulnerable and may develop insecure attachment styles even in only moderately stressful childhood environments^60^. Indeed, quality of interpersonal relationships is protective against psychopathology in adulthood for individuals exposed to childhood abuse^61^, which suggests that there could be complex bidirectional causality, such that early life adversity partially determines attachment style, which then partially determines resilience or vulnerability to such adversity. In our sample we found individuals in all four quadrants of the ASQ/CTQ graph, although overall there was a moderate positive association between early life adversity and insecure adult attachment style. The finding that genetic factors can affect adult attachment style^37,62^ implies that attachment style in adults is a result of an interaction between congenital and environmental influences.

Reproductive depression has been hypothesized to represent a distinct clinical entity that is separate from nonreproductive depression^63^. Based on our finding that attachment insecurity has a uniquely high association with depression in the perinatal period, we suggest that the association with insecure attachment style and difficult role transition is a major factor that distinguishes perinatal depression from nonreproductive depression.

We acknowledge that perinatal depression is a multifactorial disorder^64,65^, and that distinct vulnerabilities may correspond to the subtypes of the disorder. Previous authors have reported five different trajectories of depression across the perinatal period^66^, with distinct predisposing factors associated with each trajectory. Of interest, this group identified childhood trauma, intimate partner violence, and single parenthood or poor partner support as associated with all perinatal depressive trajectories in comparison to the healthy trajectory. This emphasizes the importance of life adversity and interpersonal relationship factors in predisposition to perinatal depression.

### Broad DNA methylation signature across the capture region

We identified broad changes in DNA methylation in association with early life adversity in buccal epithelial cells, a result that has been reported by others as well^20^. In contrast, we found far fewer results of significance for perinatal depression and for attachment insecurity. This is consonant with the theory that early trauma may have global and durable effects on organismal physiology (allostatic load), carried out in part by epigenomic modifications to the DNA blueprint, and detectable in peripheral cells. The same does not appear to be true for perinatal depression or insecure attachment style. These may be more neuronally specific phenotypes and thus have much smaller methylation signatures in peripheral cells. Based on our telomere analysis, we did not detect evidence of increased allostatic load in women with either attachment insecurity or perinatal depression. This suggests that increased allostatic load may not be the major causative pathway between childhood trauma and perinatal depression.

We did find one region of the genome where methylation density was positively associated with both postpartum depression and childhood trauma. The associated gene was *PLEKHA7*, which codes for an adherens junction protein that stabilizes cadherins^67^ and nectins^68^, and has been hypothesized to orchestrate the membrane organization and function of proteins regulating calcium homeostasis^68^. *PLEKHA7* does not have any documented function specific to the perinatal period. It is possible that alterations in cell-cell communication effected by changes in transcription or expression of *PLEKHA7* could be markers of risk for depression more generally, and for perinatal depression only as a special case of this broader association.

### Single-gene DNA methylation signature within OXTR

We found more suggestive evidence for our second hypothesized pathway to perinatal depression, that of confusion in the role transition to parenthood engendered by insecure attachment style. The temporal dependence of the role of attachment style in depression risk, with the strongest association being observed in late pregnancy and the early postpartum, and a weaker association after about 4 months postpartum, is unusual and suggests that attachment insecurity may carry unique risks for depression in the perinatal period as opposed to other life epochs.

Analysis of methylation patterns within *OXTR* dovetailed with these clinical observations. Similar patterns of methylation density were observed in association with perinatal depression and with insecure attachment style, and the inverse pattern with secure attachment style, whereas the pattern of methylation density within *OXTR* that was associated with childhood trauma was distinct. This suggests that *OXTR* specifically could be involved in the association between insecure attachment and perinatal depression, although methylation patterns in this gene were not sufficiently tightly clustered for it to be identified in our screen.

The oxytocin receptor has very well-established roles in interpersonal relationships and mother-infant bonding^69,70^. Oxytocin signaling is of particular importance to the promotion of mother-infant bonding in the early postnatal period, which is the same time period in which attachment insecurity assumes a uniquely strong association with depressive symptoms. Other authors have also reported roles for DNA methylation of the oxytocin receptor in risk for postpartum depression^27–30^. Additionally, oxytocin receptor gene polymorphisms have been shown to affect adult attachment insecurity^37^. While attachment representations in new mothers are stable over time^71^, stimulated oxytocin signaling is increased in the postnatal period in association with lactation, most strongly so in the first three months postpartum^72^. If oxytocin signaling were a mediator of the association between insecure attachment style and perinatal depression, this could help explain the observed temporal specificity of the association.

Statistical testing did not support mediation of the association between insecure attachment style and perinatal depression by *OXTR* methylation. This indicates that *OXTR* methylation at this site alone is not the major mechanism for the association of these clinical variables. Nonetheless, oxytocin signaling could still be one of a number of cellular mechanisms involved in the pathway from attachment insecurity to perinatal depression, which could in combination mediate the effect. Alternatively, *OXTR* methylation may be independently associated with both perinatal depression and attachment insecurity while not being directly involved in the association between them.

## Strengths and limitations

This study is limited by small and relatively homogeneous sample and lack of a replication group. Strengths of the study include multi-time-point follow-up revealing the temporal dynamics of mood over the perinatal period, the use of next-generation sequencing, and the novel statistical and theoretical approach. Our statistical method, which privileges regions where methyl groups are found on multiple contiguous CpGs, runs the risk of overlooking regions where a single or very small group of contiguous CpG dinucleotides has functional importance; at the same time, detection is improved over methods that rely heavily on demonstrated statistical significance at single CpGs.

## Clinical Implications

Based on this research, we propose that assessments of both childhood adversity and insecure attachment style should be included more explicitly in both the evaluation and treatment planning for perinatal psychiatric patients. As current tools for assessing attachment style are somewhat unwieldy and not appropriate for screening or for clinical use, the development of more clinically useful measurement tools would be a great service to the field. As regards treatment, skilled clinicians have long known about the importance of interpersonal relationships to perinatal depression, and interpersonal psychotherapy (IPT)^13^, a treatment modality with established utility in perinatal depression, places a strong central focus on the analyses and optimization of interpersonal relationships. However many of the more recent interventions, particularly the newer remotely administered or computer-assisted therapies, lack this focus.

More generally, we propose that wider dissemination of this information and its explicit incorporation into educational programs for the next generation of perinatal mental health workers will be an important service to this patient population.

## Conclusions

We hypothesize that attachment schemas may be activated in the early postpartum period, via a biological mechanism that proceeds in part via oxytocin signaling, and specifically via alterations to the transcription of *OXTR* and other genes that are effected by highly specific patterns of methyl group deposition. Our results suggest less relevance for allostatic load engendered by childhood trauma in the causative pathway for perinatal depression.

More generally, our work supports the idea that a targeted, detailed, complex epigenetic coding pattern can be associated with specific psychiatric phenotypes, and that these complex patterns may reflect reliably observed clinical and psychological variables.

## Supplementary information

Supplementary Table 1

Supplementary Table 2

Supplementary Table 3

Supplementary Table 4

Supplementary Figure 1

Supplementary Figures 2 and 3
