# Supplementary material for: Epigenetic signatures of attachment insecurity and childhood adversity provide evidence for role transition in the pathogenesis of perinatal depression
Source: Transl Psychiatry. 2020 Feb 3;10:48. doi: 10.1038/s41398-020-0703-3 (PMC7026105; doi:10.1038/s41398-020-0703-3)
Supplement: Supplementary file 1 — Supplementary Table 1 [file 41398_2020_703_MOESM1_ESM.docx]

Supplementary Table 1. Multiple regression showing attachment insecurity and childhood trauma as predictors of depressive symptom severity.

Dependent variable: EPDS in third trimester

|  |  |  |  |  |  |  | **Parameter Estimate** | **Standard Error** | **t Value** | **Pr > \|t\|** |
| --- | --- | --- | --- | --- | --- | --- | --- | --- | --- | --- |
| **Source** | **DF** | **Type I SS** | **Mean Square** | **F Value** | **Pr > F** | **Intercept** | -3.657 | 3.191 | -1.15 | 0.255 |
| **ASQ** | 1 | 798.339 | 798.339 | 45.07 | <.0001 | **ASQ** | 0.116 | 0.043 | 2.71 | 0.008 |
| **CTQ** | 1 | 65.498 | 65.498 | 3.70 | 0.058 | **CTQ** | 0.124 | 0.083 | 1.49 | 0.140 |
| **ASQ *CTQ** | 1 | 9.718 | 9.718 | 0.55 | 0.461 | **ASQ *CTQ** | <0.001 | <0.001 | -0.74 | 0.461 |

Dependent variable: mean EPDS over months 1-3 postpartum

| **Source** | **DF** | **Type I SS** | **Mean Square** | **F Value** | **Pr > F** |  | **Parameter Estimate** | **Standard Error** | **t Value** | **Pr > \|t\|** |
| --- | --- | --- | --- | --- | --- | --- | --- | --- | --- | --- |
|  |  |  |  |  |  | **Intercept** | 0.433 | 3.187 | 0.14 | 0.892 |
| **ASQ** | 1 | 701.114 | 701.114 | 39.81 | <.0001 | **ASQ** | 0.071 | 0.043 | 1.64 | 0.104 |
| **CTQ** | 1 | 47.166 | 47.166 | 2.68 | 0.105 | **CTQ** | 0.028 | 0.083 | 0.33 | 0.742 |
| **ASQ *CTQ** | 1 | 2.889 | 2.889 | 0.16 | 0.687 | **ASQ *CTQ** | <0.001 | <0.001 | 0.40 | 0.687 |
