# Supplementary material for: Epigenetic signatures of attachment insecurity and childhood adversity provide evidence for role transition in the pathogenesis of perinatal depression
Source: Transl Psychiatry. 2020 Feb 3;10:48. doi: 10.1038/s41398-020-0703-3 (PMC7026105; doi:10.1038/s41398-020-0703-3)
Supplement: Supplementary file 3 — Supplementary Table 3 [file 41398_2020_703_MOESM3_ESM.docx]

Supplementary Table 3. Biological processes enriched among genes containing regions where methylation density is significantly associated with childhood adversity score. (N=54)

| Biological process | All  genes | Observed  # genes | Expected  # genes | fold enrichment | raw p-value | FDR |
| --- | --- | --- | --- | --- | --- | --- |
| actin cytoskeleton organization | 179 | 22 | 8.4 | 2.62 | 1.05 E-04 | 2.36 E-02 |
| cytoskeleton organization | 226 | 26 | 10.6 | 2.45 | 7.94 E-05 | 2.04 E-02 |
| transcription by RNA polymerase II | 1011 | 75 | 47.43 | 1.58 | 2.09 E-04 | 4.18 E-02 |
| transcription, DNA-templated | 1251 | 92 | 58.69 | 1.57 | 5.37 E-05 | 1.61 E-02 |
| gene expression | 1842 | 129 | 86.42 | 1.49 | 1.04 E-05 | 6.21 E-03 |
| macromolecule metabolic process | 2419 | 161 | 113.48 | 1.42 | 1.24 E-05 | 5.55 E-03 |
| organic substance metabolic process | 3338 | 213 | 156.6 | 1.36 | 4.75 E-06 | 4.27 E-03 |
| metabolic process | 4072 | 247 | 191.03 | 1.29 | 1.93 E-05 | 6.93 E-03 |
