# Supplementary material for: Epigenetic signatures of attachment insecurity and childhood adversity provide evidence for role transition in the pathogenesis of perinatal depression
Source: Transl Psychiatry. 2020 Feb 3;10:48. doi: 10.1038/s41398-020-0703-3 (PMC7026105; doi:10.1038/s41398-020-0703-3)
Supplement: Supplementary file 4 — Supplementary Table 4 [file 41398_2020_703_MOESM4_ESM.docx]

Supplementary Table 4. All genes identified where methylation density is significantly associated with either insecure attachment style, antenatal depression, or postnatal depression, with Panther molecular function classifications. (N=54)

|  | Chr | CpGs | P | FDR | Pearson coefficient | Annotation | Dist to TSS | Gene name | Gene type | Gene description | Panther functional class | |
| --- | --- | --- | --- | --- | --- | --- | --- | --- | --- | --- | --- | --- |
| Attachment Insecurity | 13 | 48 | 3.77 x10^-5^ | 5.42 x10^-5^ | -0.52 | promoter-TSS | -498 | *FGF14* | protein coding | fibroblast growth factor 14 | | growth factor |
|  | 13 | 21 | 5.42 x10^-5^ | 5.42 x10^-5^ | 0.51 | intergenic | -1,035 | *RGCC* | protein coding | regulator of cell cycle | | cell cycle regulator |
|  | Unk* | 13 | 6.47 x10^-3^ | 6.47 x10^-3^ | -0.36 | -- | -- | Similar to *DUX4L18* | pseudogene | double homeobox 4 like 18 | | -- |
| Antenatal depression | 16 | 15 | 1.23 x10^-5^ | 1.23 x10^-5^ | 0.55 | intron | 307 | *PLA2G15* | protein coding | phospholipase A2, group XV | | acyltransferase phospholipase |
| Postnatal depression | 6 | 14 | 1.73 x10^-5^ | 2.34 x10^-5^ | 0.54 | promoter-TSS | -299 | *DST* | protein coding | dystonin | | Inter- mediate filament binding |
|  | 6 | 18 | 2.34 x10^-5^ | 2.34 x10^-5^ | 0.53 | intergenic | -5,570 | *RNU7-26P* | snRNA | RNA, U7 small nuclear 26 pseudogene | | -- |
|  | 11 | 44 | 7.06 x10^-5^ | 7.06 x10^-5^ | 0.51 | intron | -44,062 | *PLEKHA7* | protein coding | pleckstrin homology domain containing, family A member 7 | | binding |
|  | 11 | 15 | 6.79 x10^-5^ | 7.06 x10^-5^ | 0.51 | promoter-TSS | 79 | *NAALAD2* | protein coding | N-acetylated alpha-linked acidic dipeptidase 2 | | metallo- protease |
|  | 13 | 8 | 9.52x10^-6^ | 9.52 x10^-6^ | 0.55 | promoter-TSS | -3 | *MBNL2* | protein coding | muscleblind-like splicing regulator 2 | | DNA binding |

*Homologous region, mapping inconclusive

|  | Chr | CpGs | P | FDR | Annotation | Dist to TSS | Gene name | Gene type | Gene description | Panther functional class |
| --- | --- | --- | --- | --- | --- | --- | --- | --- | --- | --- |
| History of  Mood Disorder | 1 | 12 | 4.2x10^-14^ | 1.4x10^-5^ | exon | 339660 | *NTNG1* | protein coding | netrin-G1, axon guidance | extracellular matrix linker protein,  receptor |
|  | 2 | 9 | 4.5x10^-14^ | 0.00017 | promoter-TSS | 708 | *FAM240C-202* | Lnc-RNA | - | - |
|  | 3 | 11 | 4.5 x10^-14^ | 4.4x10^-06^ | intron | -74035 | *STAC* | protein coding | SH3 and cysteine-rich domain-containing protein | - |
|  | 5 | 7 | 4.7x10^-14^ | 0.0019 | intergenic | 94732 | C5orf64 | protein coding | uncharacterized, secreted | - |
|  | 8 | 13 | 5.5x10^-13^ | 0.0057 | promoter-TSS | -1312 | *GSR* | protein coding | glutathione-disulfide reductase, metabolic, oxidative stress | dehydrogenase  oxidase  reductase |
|  | 9 | 7 | 6.5x10^-14^ | 0.028 | promoter-TSS | -1831 | *FOXE1* | protein coding | forkhead box E1, transcription, development | DNA binding protein |
|  | 16 | 20 | 4.4x10^-14^ | 1.9x10^-5^ | promoter-TSS | -298 | AC106738.2 | Lnc-RNA | - | - |
|  | 17 | 18 | 4.4x10^-14^ | 7.4x10^-6^ | exon | 3094 | *HIC1* | protein coding | HIC ZBTB transcriptional repressor: apoptosis, development | KRAB box transcription factor |
|  | 20 | 16 | 3.1 x10^-8^ | 0.024 | intron | -107461 | *CDH22* | protein coding | cadherin-22, cell adhesion and communication | - |
