# Supplementary figures and images for: Epigenetic signatures of attachment insecurity and childhood adversity provide evidence for role transition in the pathogenesis of perinatal depression
Source: Transl Psychiatry. 2020 Feb 3;10:48. doi: 10.1038/s41398-020-0703-3 (PMC7026105; doi:10.1038/s41398-020-0703-3)

Supplementary Figure 1. Study Flowchart

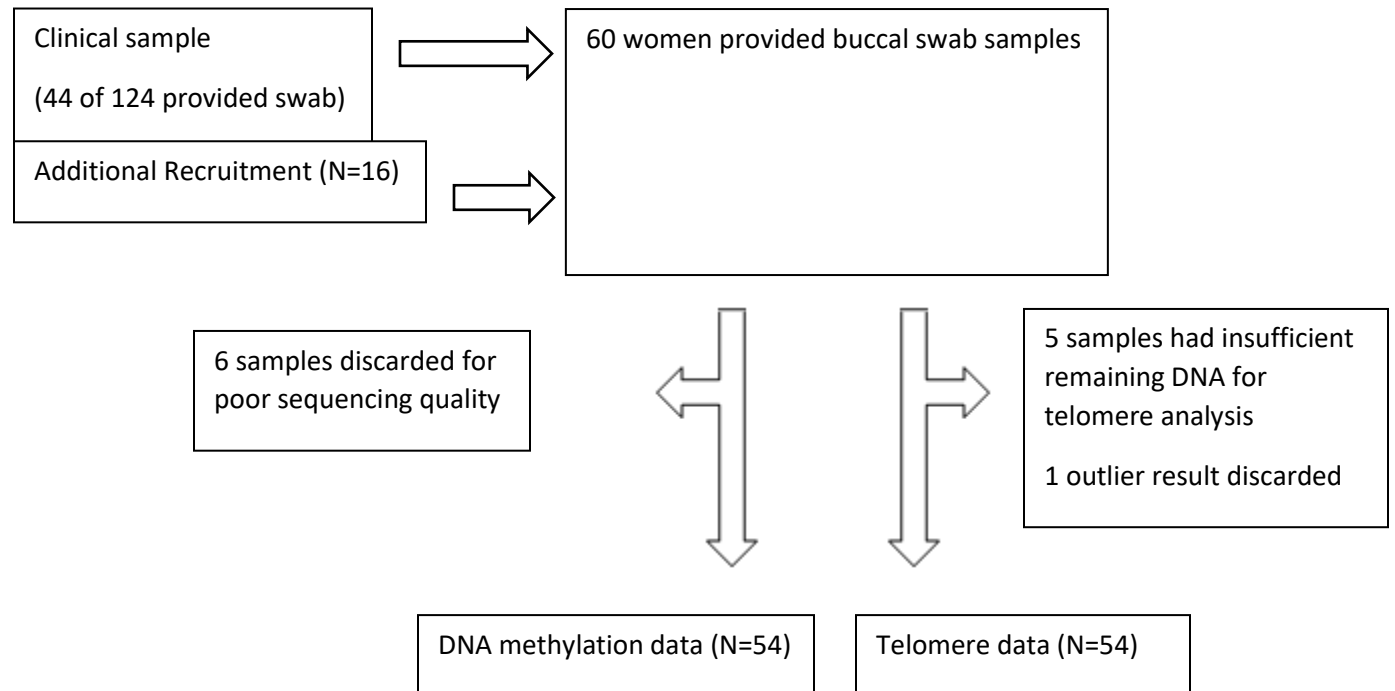

Supplement: Supplementary file 5 — Supplementary Figure 1 [file 41398_2020_703_MOESM5_ESM.pdf]
