# Supplementary material for: Epigenetic signatures of attachment insecurity and childhood adversity provide evidence for role transition in the pathogenesis of perinatal depression
Source: Transl Psychiatry. 2020 Feb 3;10:48. doi: 10.1038/s41398-020-0703-3 (PMC7026105; doi:10.1038/s41398-020-0703-3)

Supplementary Figures

Supplementary Figure 2. Association of methylation density at *PLEKHA7* intronic region with, respectively, childhood adversity score and postpartum depression severity. *PLEKHA7* methylation density vs postpartum EPDS score: Pearson correlation = 0.542, p<0.001. *PLEKHA7* methylation density vs CTQ score: Pearson correlation = 0.493, p<0.001. (N=54)


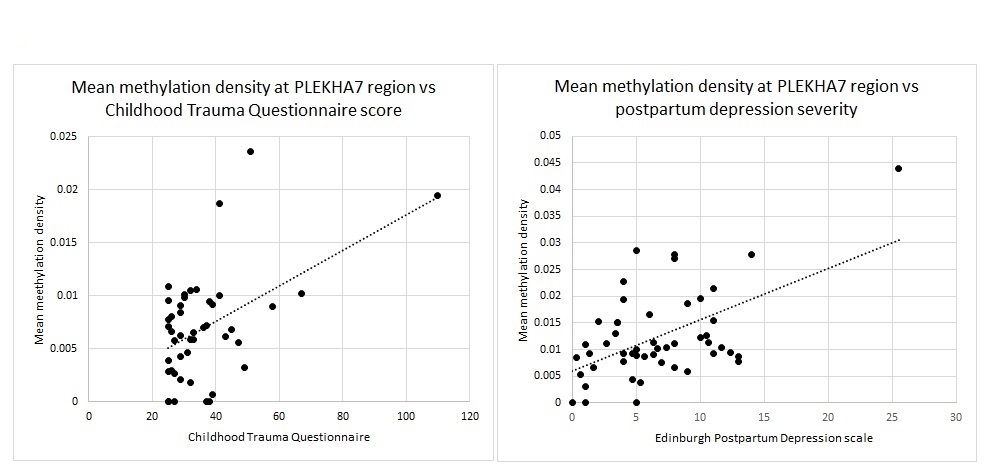


Supplementary Figure 3. Telomere analysis (N=54). Telomere length was significantly negatively associated with history of emotional abuse (ρ = -0.296, p = 0.048) and physical abuse (ρ = -0.297, p = 0.048) in childhood. Trends for shorter telomere length with greater adversity were found for other CTQ subscales and total CTQ, but did not reach statistical significance (top row). Telomere length was also positively associated with educational attainment (p=0.004, data not shown). Telomere length was not associated with attachment insecurity or depressive symptoms (bottom row).


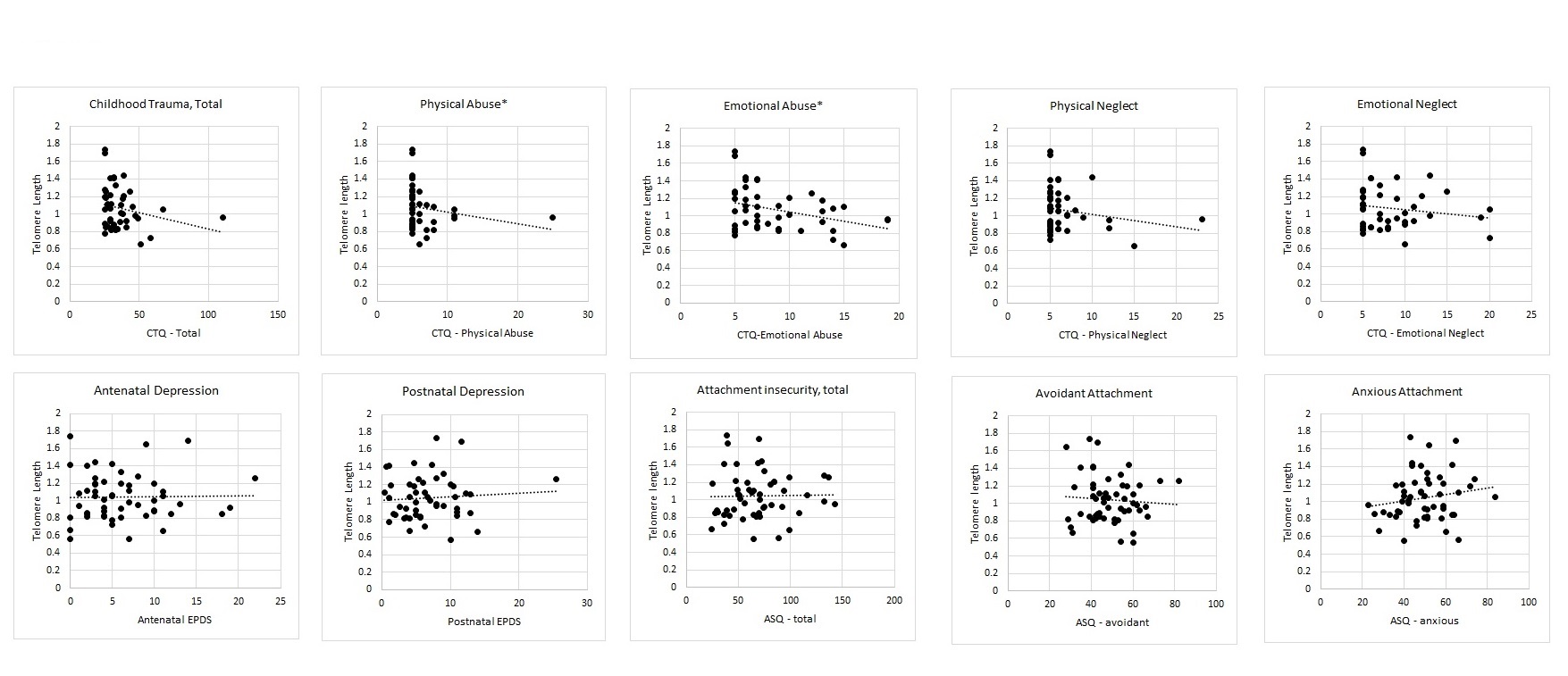

Supplement: Supplementary file 6 — Supplementary Figures 2 and 3 [file 41398_2020_703_MOESM6_ESM.docx]
